# Identification of Two Regulators of Virulence That Are Conserved in *Klebsiella pneumoniae* Classical and Hypervirulent Strains

**DOI:** 10.1128/mBio.01443-18

**Published:** 2018-08-07

**Authors:** Michelle Palacios, Taryn A. Miner, Daniel R. Frederick, Victoria E. Sepulveda, Joshua D. Quinn, Kimberly A. Walker, Virginia L. Miller

**Affiliations:** a Department of Microbiology and Immunology, University of North Carolina, Chapel Hill, North Carolina, USA; b Department of Genetics, University of North Carolina, Chapel Hill, North Carolina, USA; Emory University School of Medicine

**Keywords:** EmrR, KvrA, KvrB, MarR, ST258, SlyA, capsule, hypermucoviscosity

## Abstract

Klebsiella pneumoniae is widely recognized as a pathogen with a propensity for acquiring antibiotic resistance. It is capable of causing a range of hospital-acquired infections (urinary tract infections [UTI], pneumonia, sepsis) and community-acquired invasive infections. The genetic heterogeneity of K. pneumoniae isolates complicates our ability to understand the virulence of K. pneumoniae. Characterization of virulence factors conserved between strains as well as strain-specific factors will improve our understanding of this important pathogen. The MarR family of regulatory proteins is widely distributed in bacteria and regulates cellular processes such as antibiotic resistance and the expression of virulence factors. *Klebsiella* encodes numerous MarR-like proteins, and they likely contribute to the ability of K. pneumoniae to respond to and survive under a wide variety of environmental conditions, including those present in the human body. We tested loss-of-function mutations in all the *marR* homologues in a murine pneumonia model and found that two (*kvrA* and *kvrB*) significantly impacted the virulence of K1 and K2 capsule type hypervirulent (*hv*) strains and that *kvrA* affected the virulence of a sequence type 258 (ST258) classical strain. In the *hv* strains, *kvrA* and *kvrB* mutants displayed phenotypes associated with reduced capsule production, mucoviscosity, and transcription from *galF* and *manC* promoters that drive expression of capsule synthesis genes. In contrast, *kvrA* and *kvrB* mutants in the ST258 strain had no effect on capsule gene expression or capsule-related phenotypes. Thus, KvrA and KvrB affect virulence in classical and *hv* strains but the effect on virulence may not be exclusively due to effects on capsule production.

## IMPORTANCE

In addition to having a reputation as the causative agent for hospital-acquired infections as well as community-acquired invasive infections, Klebsiella pneumoniae has gained widespread attention as a pathogen with a propensity for acquiring antibiotic resistance. Due to the rapid emergence of carbapenem resistance among K. pneumoniae strains, a better understanding of virulence mechanisms and identification of new potential drug targets are needed. This study identified two novel regulators (KvrA and KvrB) of virulence in K. pneumoniae and demonstrated that their effect on virulence in invasive strains is likely due in part to effects on capsule production (a major virulence determinant) and hypermucoviscosity. KvrA also impacts the virulence of classical strains but does not appear to affect capsule gene expression in this strain. KvrA and KvrB are conserved among K. pneumoniae strains and thus could regulate capsule expression and virulence in diverse strains regardless of capsule type.

## INTRODUCTION

Klebsiella pneumoniae is a Gram-negative bacterium capable of causing a wide range of infections such as urinary tract infections (UTI), sepsis, liver abscesses, and pneumonia (1–6). Capsule, lipopolysaccharide (LPS), adhesion factors, and siderophores frequently emerge as the primary K. pneumoniae virulence determinants, with capsule being the most extensively studied. Currently, 134 capsule types have been identified in K. pneumoniae (7). One trait strongly associated with virulence in *Klebsiella* is the overproduction of capsule, which contributes to a hypermucoviscosity (HMV) phenotype (5, 8). HMV strains are “string test” positive and tend to be hypervirulent (*hv*) (8, 9). Our relatively limited knowledge of conserved virulence determinants and the high diversity of surface polysaccharides pose challenges for developing vaccines and new therapeutics (10–12).

While first characterized for its role in antibiotic resistance in Escherichia coli (13, 14), the MarR (multiple antibiotic resistance regulator) family of transcriptional regulators is known to regulate the expression of genes encoding proteins involved in metabolic pathways, stress responses, and virulence factors (14–20). These proteins are characterized by a winged helix-turn-helix DNA binding domain and can both positively and negatively affect gene expression (21, 22). Transcriptional regulation by these proteins often results in modifications of the bacterial cell surface (23), and several MarR family members have been linked to virulence in the *Enterobacteriaceae*. In *Salmonella*, SlyA regulates *Salmonella* pathogenicity island-2 genes and contributes to resistance to oxidative stress, bacterial survival within macrophages, and bacterial survival in a murine model of infection (17, 18, 23–26). RovA, a member of the MarR/SlyA family, regulates expression of *inv* (an adhesion and invasion factor) in the enteric pathogens Yersinia enterocolitica and Yersinia pseudotuberculosis (27–29) as well as expression of the *psa* locus of Yersinia pestis, the causative agent of bubonic and pneumonic plague (30).

An *in silico* comparative study found the copy number of *marR*-like genes to range from 2 to 11 within the *Enterobacteriaceae*, with an average of 5.9 genes (22). That same study identified some K. pneumoniae strains that have as many as 11 *marR* homologues (22). We hypothesize that this high number of *marR-*like genes contributes to the ability of K. pneumoniae to survive in a wide variety of environments, including the human host, and that a subset of these genes regulates expression of virulence phenotypes.

In this report, we describe the contribution of the MarR family to K. pneumoniae virulence in a murine pneumonia model. Strain KPPR1S (31), a derivative of ATCC 43816, was found to contain nine *marR*-like genes (32). We constructed insertion disruption mutations in each of the *marR* homologues of strain KPPR1S and tested them in our pneumonia model. Two of these genes, designated *kvrA* and *kvrB*, affected virulence. The impact of KvrA and KvrB on virulence in this *hv* strain is likely due at least in part to their effect on expression of capsule genes and the HMV phenotype. Importantly, these roles were conserved in another *hv* K. pneumoniae strain that produces a different capsule type, and *kvrA* is required for full virulence of a sequence type 258 (ST258) classical strain.

## RESULTS

### Two *marR-*like genes contribute to K. pneumoniae virulence.

**FIG 1:**
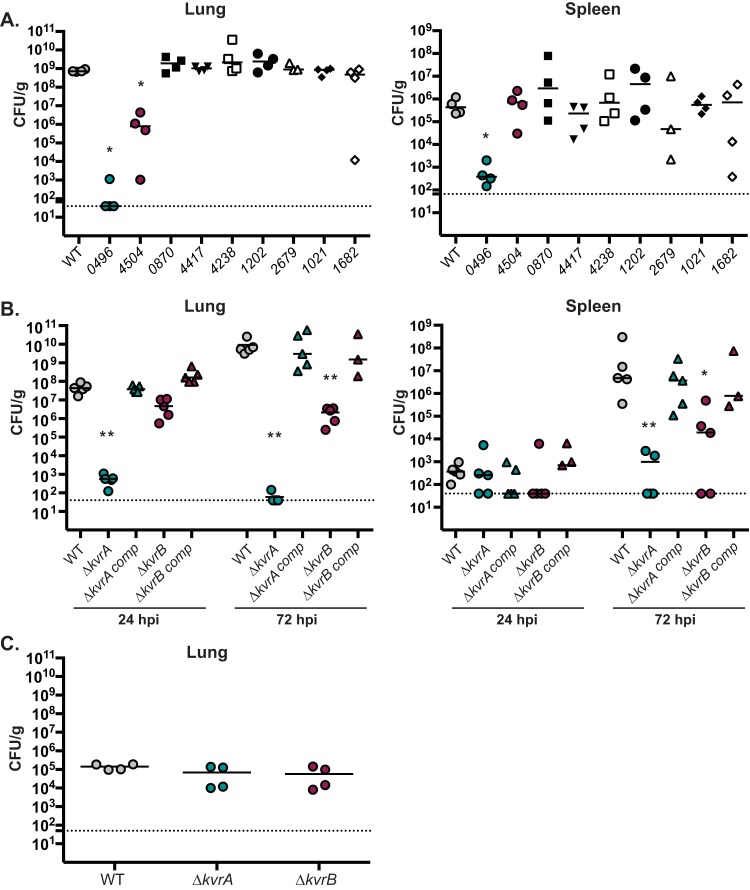
Members of the *marR* family contribute to virulence in a mouse model of pneumonia. Mice were inoculated i.n. with 2 × 10^4^ CFU of the indicated strains. At the indicated times, mice were euthanized, and the lungs and spleens were homogenized and plated for bacterial enumeration. Each symbol represents one mouse. The dotted line indicates the limit of detection, and symbols on the dotted line indicate CFU counts that were below the limit of detection. These data are from an individual representative experiment. The Mann-Whitney test was used for statistical analyses comparing each mutant to the WT. *, *P* < 0.05; **, *P* < 0.01. (A) Single-time-point (48 hpi) analyses of screen *marR* family mutants for virulence defects. (B) Kinetic analyses of the WT, Δ*kvrA* (VK277), Δ*kvrB* (VK410), *kvrA* complemented (VK278), and *kvrB* complemented (VK417) strains. (C) Single-time-point (90 min postinfection [mpi]) analyses of the WT, Δ*kvrA* (VK277), and Δ*kvrB* (VK410) strains.

The MarR family of transcriptional regulators has been implicated in virulence in several members of the *Enterobacteriaceae* (17, 21, 24, 30, 33, 34). *Klebsiella* species contain more than the average number of *marR*-like genes, and we hypothesize that some members of this family are important for adaptation of K. pneumoniae in the mammalian host. Thus, we screened the genome of KPPR1S (wild-type [WT] strain) for putative *marR* genes and constructed loss-of-function mutants for each of the nine *marR* homologues identified. Growth curves (determined using optical density at 600 nm [OD_600_] and CFU counts per milliliter) indicated that none of the mutants displayed a growth defect *in vitro* in Luria-Bertani (LB) medium (data not shown). To assess the effects on virulence, mice were intranasally (i.n.) infected with the WT strain or with each mutant individually and sacrificed at 48 h postinoculation (hpi) for bacterial enumeration. Two mutants, VK055_0496 and VK055_4504, displayed a decrease in bacterial burden in the lungs of infected mice compared to WT-infected mice (Fig. 1A). The spleens of mice infected with the WT had nearly 10^6^ CFU/g of tissue, while the VK055_0496 mutant was barely detectable (Fig. 1A).

On the basis of our initial screen, we determined that VK055_0496 and VK055_4504 are important for infection of the lung and named these regulators KvrA and KvrB (*Klebsiella* virulence regulator), respectively. Further kinetics experiments using in-frame deletion mutants of *kvrA* (VK277) and *kvrB* (VK410) indicated that at 24 hpi, the Δ*kvrA* mutant was barely detectable in the lung whereas mice infected with the Δ*kvrB* mutant had ~1-log-lower levels of CFU/g than the WT. By 72 hpi, the bacterial burdens of mice infected with the WT had increased several logs, but the Δ*kvrA* mutant was undetectable, and the bacterial burden of the Δ*kvrB* mutant remained comparable to that seen at 24 hpi (Fig. 1B). The spleens of Δ*kvrA* mutant*-*infected mice had few recoverable CFU at either 24 or 72 hpi, while the mice infected with the Δ*kvrB* mutant had splenic burdens that were more than 2 logs lower than the levels seen with the mice infected with the WT at 72 hpi (Fig. 1B). To test if the survival defect of the mutants was due to an inability to reach the lung, mice were infected with the WT or Δ*kvrA* or Δ*kvrB* strain and the lungs were harvested at 90 min postinoculation. The three strains displayed comparable bacterial burdens in the lungs at that early time point, suggesting that the ability of the mutants to initially reach the lungs was not impacted (Fig. 1C). Subsequent analysis indicated that the virulence defect of the Δ*kvrA* and Δ*kvrB* mutants could be attributed to the loss of KvrA and KvrB, as demonstrated by complementation of the Δ*kvrA* and Δ*kvrB* mutants (Fig. 1B). Together, these results demonstrate that KvrA and KvrB contributed to K. pneumoniae virulence in a lung model of infection.

### The Δ*kvrA* and Δ*kvrB* mutants induced an altered immune response.

**FIG 2:**
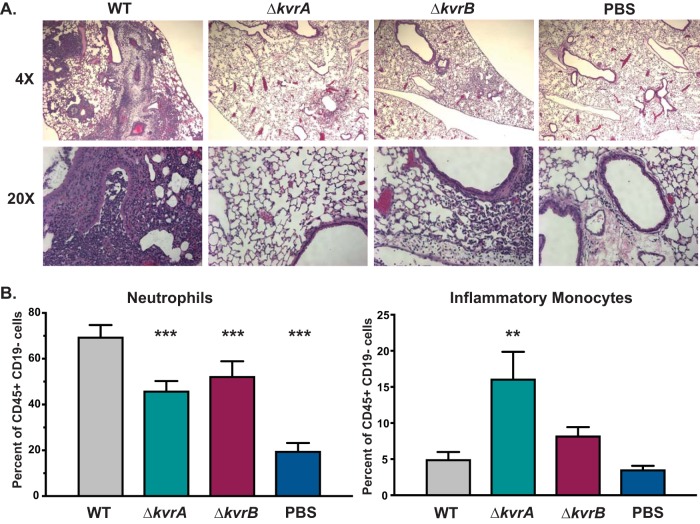
Innate immune cell infiltration during K. pneumoniae infection. (A) H&E staining of mouse lungs inoculated with the WT strain, the Δ*kvrA* (VK277) mutant, or the Δ*kvrB* (VK410) mutant or PBS at 72 hpi. (B) Lungs were processed 24 hpi and evaluated by flow cytometry to identify innate immune cell populations. These data are from an individual representative experiment. Two-way ANOVA tests were performed for statistical analysis. **, *P* < 0.01; ***, *P* < 0.001.

Infection with K. pneumoniae is known to induce a significant inflammatory response marked by lung lesions and neutrophil infiltration (35–37). Neutrophils contribute to bacterial clearance of some K. pneumoniae strains but not others (38, 39). Neutrophils are considered important for clearance of ATCC 43816 (i.e., KPPR1S); however, high numbers of neutrophils also may be detrimental to host health (39). Inflammatory monocytes also are considered a primary cell type functioning in defense against K. pneumoniae (38). Consistent with the reduced number of CFU in the lung, infection of mice with the Δ*kvrA* and Δ*kvrB* mutants resulted in less inflammation than infection with the WT at 72 hpi (Fig. 2A). To more carefully examine the histological changes following infection, the lungs from mice inoculated with the WT or Δ*kvrA* or Δ*kvrB* strain or subjected to mock inoculation (1× phosphate-buffered saline [PBS]) were processed at 24 hpi for flow cytometric analysis. Infection with all three strains resulted in a significant increase in levels of neutrophils compared to the mock infection results (Fig. 2B). Although the infections with the two mutants resulted in elevated levels of neutrophils compared to the mock infection results, the increase in the levels of neutrophils was significantly lower than that seen with the WT. Among the cell populations examined, there was a 3-fold increase in the percentage of inflammatory monocytes in the mice infected with the Δ*kvrA* mutant compared to the mice infected with the WT (Fig. 2B). Inflammatory monocytes have been implicated in protection from K. pneumoniae (38), and the higher proportion of this cell type may have contributed to the rapid clearance of the Δ*kvrA* mutants.

### The Δ*kvrA* and Δ*kvrB* mutants showed increased associations with murine BMDMs.

**FIG 3:**
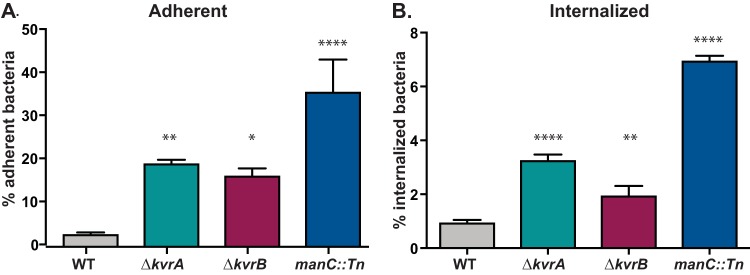
Mutations in *kvrA* or *kvrB* affect adherence and uptake of K. pneumoniae in BMDMs. BMDMs were inoculated with the WT strain or Δ*kvrA* (VK277), Δ*kvrB* (VK410), or *manC*::*Tn* (VK060) mutant strain at an MOI of 50. Adherence (A) and internalization (B) were measured as described in Materials and Methods. One-way ANOVA tests were performed for statistical analyses comparing the WT strain to the indicated mutants. *, *P* < 0.05; **, *P* < 0.01; ****, *P* < 0.0001.

Because of the reduced bacterial burden of the Δ*kvrA* and Δ*kvrB* mutants in mice, we wanted to determine if these strains had altered interactions with murine macrophages. The Δ*kvrA* and Δ*kvrB* mutants were inoculated onto murine bone marrow-derived macrophages (BMDMs) in culture and were assessed for adherence to and internalization into these host-derived cells. A capsule mutant, *manC* (VK60), was included as a control as it is known to be more adherent and more readily phagocytosed than the WT (40, 41). Only about 3% of the WT bacteria adhered to the BMDMs, whereas more than 35% of the *manC* mutant bacteria were cell associated (Fig. 3A). The Δ*kvrA* and Δ*kvrB* mutants were 4-to-6-fold more adherent than the WT (19% and 16%, respectively). A similar trend was observed when these strains were assayed for internalization by BMDM in a gentamicin protection assay. About 1% of the WT and 7% of the *manC* bacteria were internalized, demonstrating the antiphagocytic properties of capsule (Fig. 3B). The Δ*kvrA* and Δ*kvrB* mutants were internalized at levels of about 3% and 2%, respectively. These intermediate adherence and internalization phenotypes suggest that the Δ*kvrA* and Δ*kvrB* strains have altered interactions with host cells.

### KvrA and KvrB contribute to capsule production.

**FIG 4:**
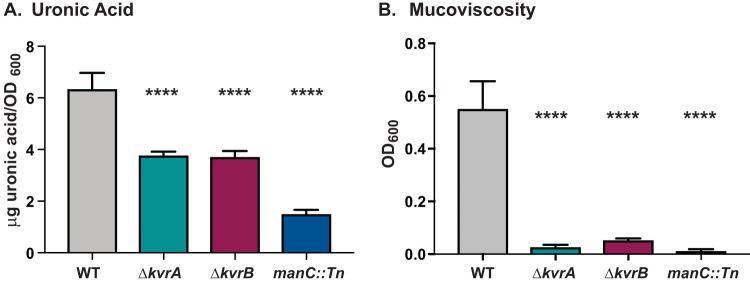
Capsule production was reduced in the Δ*kvrA* and Δ*kvrB* mutants. WT, Δ*kvrA* (VK277), Δ*kvrB* (VK410), or *manC*::*Tn* (VK060) cultures were grown in LB for 6 h and subjected to (A) uronic acid quantification and (B) mucoviscosity analysis as described in Materials and Methods. One-way ANOVA tests were performed for statistical analyses comparing the WT strain to the indicated mutants. ****, *P* < 0.0001.

During mutant construction, we observed that colonies of the Δ*kvrA* and Δ*kvrB* mutants appeared to be less mucoid and less hypermucoviscous. Thus, we hypothesized that capsule production levels would be decreased in the mutants. Glucuronic acid is a key component of many different capsules, including the K2 capsule produced by KPPR1S. Measurement of uronic acid content is therefore frequently used to quantify capsule production (42, 43). We determined the uronic acid concentrations in the Δ*kvrA* and Δ*kvrB* mutants along with the WT and the *manC* mutant. The *manC* mutant produced about 1.5 µg uronic acid/OD_600_, which is about 25% of the level produced by the WT (Fig. 4A). The Δ*kvrA* and Δ*kvrB* mutants each produced uronic acid at only ~60% of the WT levels, indicating that these mutants produce less capsule than the WT.

Mucoviscosity can be measured using a sedimentation assay (44, 45). HMV strains such as KPPR1S do not form tight pellets when centrifuged, and this can be quantified by measuring the OD_600_ of the supernatant following low-speed centrifugation. The OD_600_ of the WT was about 0.5, but the *manC* strain formed a tight pellet and the supernatant was essentially cleared, measuring 0.01 (Fig. 4B). The Δ*kvrA* and Δ*kvrB* mutants also formed tight pellets with cleared supernatants. These data, consistent with the reduced uronic acid levels, indicate that the Δ*kvrA* and Δ*kvrB* strains have reduced mucoviscosity.

### KvrA and KvrB positively regulate capsule gene expression.

**FIG 5:**
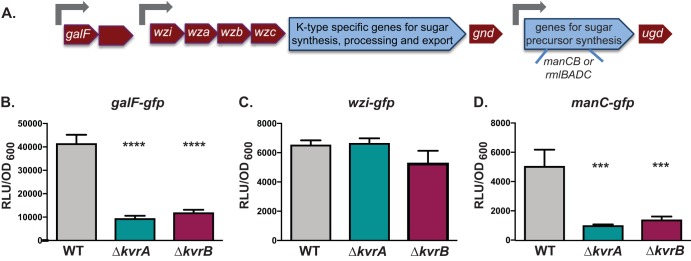
KvrA and KvrB regulate expression of capsule gene promoters. (A) Schematic of a *cps* locus. Gray arrows indicate known promoters, genes indicated in red are highly conserved between different *cps* loci, and genes indicated in blue are variable between *cps* loci. The *manC* gene is also known as *cpsB*. (B to D) Cultures of the indicated strains containing the *galF*, *wzi*, or *manC* promoter from KPPR1S cloned into pPROBE were grown in LB for 6 h. Fluorescence was measured and normalized to the culture OD_600_. These data are representative of results from an individual experiment. One-way ANOVA tests were performed for statistical analyses comparing the WT strain to the indicated mutants. ***, *P* < 0.001; ****, *P* < 0.0001.

Given the observations indicating that Δ*kvrA* and Δ*kvrB* have reduced hypermucoviscosity and produce less uronic acid than the WT, it is likely that expression of the capsule locus (*cps* locus) is reduced in these mutants. We constructed transcriptional *gfp* fusions to the known capsule promoters located upstream of *galF*, *wzi*, and *manC* (also known as *cpsB*) (43) and transformed these into the WT and mutant strains (Fig. 5A). The *wzi* promoter appeared to be affected only minimally by the loss of KvrA or KvrB. However, the levels associated with the *galF* and *manC* promoters were significantly decreased (20% to 25% of WT expression) in both the Δ*kvrA* and Δ*kvrB* mutants (Fig. 5B to D), indicating that loss of KvrA and KvrB results in a reduction in capsule synthesis gene expression.

**FIG 6:**
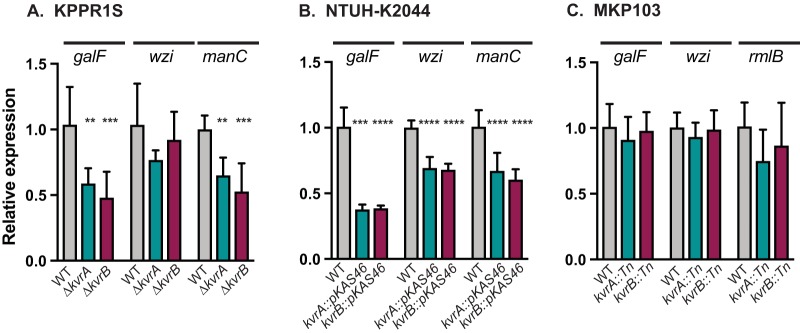
qRT-PCR analysis of *cps*-regulated genes from diverse K. pneumoniae strains. Strains were grown in LB for 2 h, and RNA was isolated and analyzed by qRT-PCR for expression of the *galF*, *wzi*, *manC*, or *rmlB* gene. Data are presented relative to each individual WT strain. (A) Analysis in KPPR1S (WT, Δ*kvrA* [VK277], and Δ*kvrB* [VK410] strains). (B) Analysis in NTUH-K2044 (WT, *kvrA*::*pKAS46* [VK606], and *kvrB*::*pKAS46* [VK607] strains). (C) Analysis in MKP103 (WT, *kvrA*::*Tn* [VK402], and *kvrB*::*Tn* [VK404] strains). One-way ANOVA tests were performed for statistical analyses comparing the WT strain to the indicated mutants. **, *P* < 0.01; ***, *P* < 0.001; ****, *P* < 0.0001.

KvrA and KvrB are found in most other K. pneumoniae strains, and thus we wanted to determine if KvrA and KvrB have conserved roles in different strains. Because of the high prevalence of the K1 serotype among *hv* isolates associated with community-acquired liver abscess infections (5, 46), we chose to test NTUH-K2044, which was isolated from a liver abscess (47). We constructed insertional mutations in *kvrA* (VK606) and *kvrB* (VK607) and performed quantitative reverse transcription-PCR (qRT-PCR) analysis on *galF*, *wzi*, and *manC* in both the NTUH-K2044 and KPPR1S backgrounds. In the KPPR1S strains, we observed that the levels of expression of the *galF* and *manC* genes in the Δ*kvrA* and Δ*kvrB* strains were significantly reduced, consistent with the *gfp* reporter data (Fig. 6A). The levels of the *galF* and *manC* genes were also significantly reduced in the NTUH-K2044 *kvrA* and *kvrB* mutants (Fig. 6B). This analysis also revealed that *wzi* expression was significantly reduced in the NTUH-K2044 mutants (Fig. 6B) and appeared to be slightly reduced in the KPPR1S mutants as well, although the results were not statistically significant (Fig. 6A). These data indicate that KvrA and KvrB regulate expression of several promoters in the *cps* locus from strains with at least two different capsule types, K1 and K2.

Both type K1 and type K2 capsules incorporate mannose, and this is true of ~73% of capsule types; most of the remaining capsule types contain rhamnose, and some contain both mannose and rhamnose (48). Sequence type 258 (ST258) strains have recently been shown to be associated with many nosocomial infections caused by carbapenem-resistant K. pneumoniae, including an outbreak at the NIH (49). One such ST258 clinical isolate, KPNIH1 (49), has a *cps* locus containing genes that produce a rhamnose subunit, and it harbors *kvrA* and *kvrB*. MKP103, a carbapenem-sensitive derivative of KPNIH1, was constructed and was used to generate an ordered library of transposon mutants (50). The effect of *kvrA* (VK402) and *kvrB* (VK404) mutations on expression of the *galF*, *wzi*, and *rmlB* genes was tested in the MKP103 strain using qRT-PCR (Fig. 6C). Interestingly, although the *galF* and *wzi* promoters are virtually identical among all three strains, KvrA and KvrB did not appear to regulate these promoters in MKP103. One possible explanation is that *kvrA* or *kvrB* is not expressed in this strain; we examined this possibility by qRT-PCR and verified that both genes are indeed expressed (data not shown). Thus, these data suggest that the roles of KvrA and KvrB in regulating expression of *galF* and *wzi* expression are indirect. The *rmlB* promoter is quite different from the *manC* promoter, and KvrA and KvrB did not appear to regulate this promoter in MKP103.

**FIG 7:**
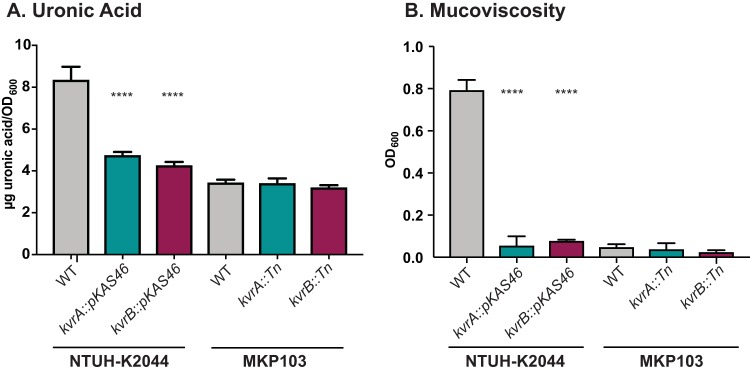
Capsule production in *kvrA* and *kvrB* mutants of NTUH-K2044 and MKP103. Assays were performed as described for Fig. 4. (A) Uronic acid quantification. (B) Mucoviscosity. The strains used were the same as those described in the Fig. 6 legend. One-way ANOVA tests were performed for statistical analyses comparing the WT strain to the indicated mutants. ****, *P* < 0.0001.

In addition to capsule regulation, we analyzed capsule production and mucoviscosity of the NTUH-2044 and MKP103 strains. As with KPPR1S, we found that uronic acid content was significantly reduced in both the NTUH-K2044 *kvrA* and *kvrB* mutants to ~60% of the level seen with the parental strain (Fig. 7A). Similarly, both the *kvrA* and the *kvrB* mutants of NTUH-K2044 had reduced mucoviscosity (Fig. 7B). Consistent with the qRT-PCR data, KvrA and KvrB affected neither uronic acid content nor mucoviscosity in MKP103 (Fig. 7). However, MKP103 does not produce as much capsule as either KPPR1S or NTUH-K2044 and is not HMV. Together, these data suggest that the role of KvrA and KvrB in capsule production may be conserved in *hv* strains.

### Contribution of *kvrA* and *kvrB* to virulence in NTUH-K2044 and MKP103.

**FIG 8:**
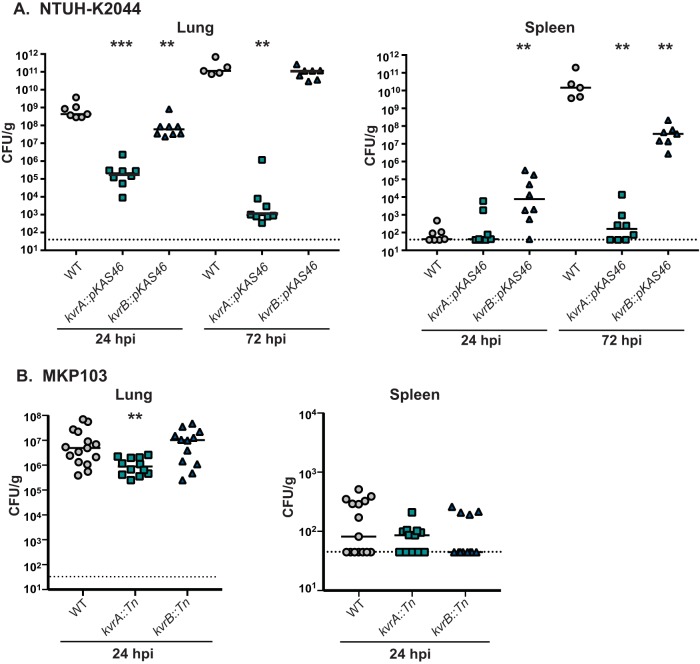
Effect of mutations in *kvrA* and *kvrB* in NTUH-K2044 or MKP103 on virulence. Mice were inoculated i.n. with the indicated strains. At various time points, mice were euthanized, and lungs (left panels) and spleens (right panels) were homogenized and plated for bacterial enumeration. Each symbol represents one mouse. The dotted line indicates the limit of detection, and symbols on the dotted line indicate CFU counts that were below the limit of detection. Mann-Whitney tests were performed for statistical analyses comparing the WT strain to the respective mutants: **, *P* < 0.01; ***, *P* < 0.001. (A) Mice were infected with 2 × 10^4^ CFU of NTUH-K2044, *kvrA*::*pKAS46* (VK606), or *kvrB*::*pKAS46* (VK607) and were sacrificed at 24 or 72 hpi. (B) Mice were infected with 2 × 10^7^ CFU of MKP103, *kvrA*::*Tn* (VK402), or *kvrB*::*Tn* (VK404) and were sacrificed at 24 hpi.

K. pneumoniae strains display significant genetic heterogeneity, and not all identified virulence factors are conserved between *Klebsiella* clinical isolates. Furthermore, the relative contributions of virulence factors to infection and pathogenesis can be dependent on the strain background (7, 48, 51, 52). However, *kvrA* and *kvrB* homologues are conserved in K. pneumoniae strains and thus could have a conserved role in virulence. We therefore tested the *kvrA* and *kvrB* loss-of-function mutants in the NTUH-K2044 and MKP103 strains in a mouse model of pneumonia. As with the KPPR1S strain, the NTUH-K2044 *kvrA* mutant was strongly attenuated in the lung and did not establish infection in the spleen (Fig. 8A). Similarly to the KPPR1S *kvrB* mutant, the NTUH-K2044 *kvrB* mutant initially colonized the lung at levels that were lower than the WT levels but was present at levels similar to the WT levels by 72 hpi (Fig. 8A). In contrast to both of the WT strains and the *kvrA* mutant, the *kvrB* mutant was detectable in the spleens of most mice by 24 hpi, and by 72 hpi the burden of the *kvrB* mutant in the spleen had increased but was still several logs lower than the WT level (Fig. 8A). These data suggest that both KvrA and KvrB contribute to the virulence of *hv* K. pneumoniae strains with different types of capsule.

Classical strains such as the ST258 strains prevalent in nosocomial outbreaks of carbapenem-resistant strains do not exhibit the same level of virulence in mouse models as the *hv* strains (39, 53). Nevertheless, when administered at a high dose (10^7^ CFU), these classical isolates can initially colonize the lungs of mice but are then cleared within 48 to 72 hpi (39, 53). Because *kvrA* and *kvrB* are part of the core K. pneumoniae genome, we assessed the virulence of the *kvrA* and *kvrB* mutants in MKP103. At 24 hpi, the *kvrB* mutant behaved similarly to the WT strain, whereas the *kvrA* mutant colonized the lungs of these mice at a significantly reduced level (Fig. 8B). Bacterial burdens in the spleen were very low for all three strains, but the *kvrA* mutant appeared to be defective for spleen colonization relative to the WT and *kvrB* strains (Fig. 8B). This trend for the *kvrA* mutant was significant (*P* < 0.05) in analyses of only those mice with detectable CFU. C57BL/6 mice were used in these studies, and it is possible that the *kvrA* mutant, as well as the *kvrB* mutant, would have a stronger phenotype in a host comparable to the immunocompromised patients that are typically infected with classical strains. Nevertheless, it appears that *kvrA* is important for the virulence of the representative *hv* and classical strains tested here.

## DISCUSSION

The MarR family of transcriptional regulators has been implicated in regulation of virulence genes in several members of the *Enterobacteriaceae* (17, 18, 21, 24, 26, 34, 54). This work examines the contribution of the MarR family to K. pneumoniae virulence by first screening loss-of-function mutations in nine *marR*-like genes in a K2 *hv* strain (KPPR1S) using a mouse model of pneumonia. Two uncharacterized transcriptional regulators, KvrA and KvrB, were found to be important for virulence in this model. The *kvrA* mutant was essentially avirulent. Inflammatory monocytes have been shown to be critical for clearance of a K. pneumoniae ST258 strain by producing tumor necrosis factor alpha (TNF-α) that then recruits interleukin-17 (IL-17)-producing innate lymphocytes (38), and IL-17 has also been shown to control infection with ATCC 43816 (55), the strain from which KPPR1S was derived. Thus, the increased number of inflammatory monocytes observed after infection with the Δ*kvrA* mutant was likely contributing to the rapid clearance of this mutant. Mutations in *kvrA* and *kvrB* in a K1 *hv* strain (NTUH-K2044) also resulted in a virulence defect in this pneumonia model. In addition, mutations in *kvrA* and *kvrB* resulted in increased adherence to and uptake of KPPR1S by murine BMDM. These phenotypes are consistent with those of capsule-deficient strains (56), and indeed, KvrA and KvrB were found to regulate capsule gene expression from the *galF* and *manC* promoters in KPPR1S and NTUH-K2044. However, *kvrA* and *kvrB* mutations in classical ST258 strain MKP103 did not affect expression from the conserved *galF* promoter. Whether these effects of KvrA and KvrB on expression from the *manC* and *galF* promoters are direct or indirect is not known. The lack of an effect on *galF* expression in the MKP103 *kvrA* and *kvrB* mutants suggests that the effect(s) on *galF* expression in the *hv* strains may be indirect, acting through a protein present only in *hv* strains.

The contribution of the MarR family to the virulence of K. pneumoniae may be both strain and infection site dependent. Previously, the role of the MarR family protein, PecS, was shown to be important for repressing the type 1 fimbrial gene locus in K. pneumoniae strain CG43 (57). Type 1 fimbriae have been implicated in binding to mannose-containing structures and are important for causing UTI (58). A high-throughput genetic screen for virulence factors that are important for a pneumonic infection found that the *pecS* mutant was attenuated only modestly in a coinfection experiment (59). The *pecS* ortholog (geneID VK055_4417) was targeted in our initial screen and found to be dispensable for virulence in the lung. This is consistent with a previous study where an *mrk* (type 3 fimbrial) mutant and an *mrk fim* double mutant were not attenuated in a lung model of *Klebsiella* infection (60) even though they were attenuated in a UTI model (61). While our study demonstrated that PecS is dispensable for KPPR1S infection in the lung, it may serve an important role in other infection sites such as the urinary tract.

The bacterial capsule serves a variety of functions, including, but not limited to, protection from the immune response of the host (56, 62–64). Capsule synthesis in K. pneumoniae is homologous to assembly of the Wzy-dependent assembly of group 1 capsules of E. coli (65). Following polymerization, the polysaccharide chains are transported across the outer membrane and are thought to be anchored to the surface via the Wzi lectin (66, 67). E. coli and K. pneumoniae are among the few gammaproteobacteria that encode Wzi proteins (67); in this study, using the sedimentation assay, a *wzi* mutant was no longer hypermucoviscous.

Our study has revealed a role for KvrA and KvrB in regulation of sugar production for capsule synthesis in type K1 and type K2 strains as determined by a reduction in expression of the *galF* and *manC* promoters and a reduction in uronic acid levels. Furthermore, hypermucoviscosity, a property associated with *hv* strains (but not classical strains) and generally associated with more abundant capsule in the literature (5, 9, 68), was significantly reduced in both the *kvrA* and *kvrB* mutants in the *hv* strains. However, it is unclear whether cell-associated capsule or released extracellular polysaccharide or both are responsible for the changes in hypermucoviscosity. The difference between surface-associated and released capsule polysaccharides may reflect a difference in function. For instance, surface-associated capsule can inhibit complement deposits and thus evade the membrane attack complex, and it can block attachment to phagocytic cells (69). Free extracellular capsule may act as a decoy to sequester antimicrobial peptides produced by the host (70). Therefore, it is possible that the reduction in uronic acid and mucoviscosity that we observed may reflect a change in the ratio of free and bacterial-surface-associated saccharides. On the basis of previous studies (67), this change may be mediated by Wzi, as we observed a modest decrease in expression from the *wzi* promoter. However, it could also be due in part to the reduction in synthesis of precursor sugars via reduced expression from the *galF* and *manC* promoters or to altered expression of unknown genes that influence mucoviscosity.

The primary sequences of the MarR/SlyA/RovA proteins are highly conserved among different bacteria; however, the degree of similarity between different MarR-family homologues can vary substantially. For instance, while KvrA shares 84% identity with SlyA of Salmonella enterica (71) and KvrB shares 93% identity with EmrR of E. coli (72), KvrA and KvrB share only 31% identity. A consensus DNA-binding sequence has been difficult to identify for many of these family members, and regulation by this class of regulators appears to be largely mediated by derepression of H-NS transcriptional silencing (73). H-NS regulates many horizontally acquired genes, and this regulation has been shown in many instances to be counteracted by regulators of the MarR family (27, 74–76). Thus, the regulons of MarR regulators can differ between closely related bacterial species and even between strains of the same species (28).

We found that KvrA and KvrB are necessary for capsule expression, and, given the comparable capsule production defects but different disease phenotypes of *kvrA* and *kvrB* mutants, we believe that KvrA and KvrB may be regulating genes in addition to the *cps* locus, and efforts to define the regulons are currently under way in our laboratory along with studies to examine direct versus indirect regulation. As less than 40% of genes are conserved across different K. pneumoniae strains (52), identifying highly conserved targets affecting growth and survival of K. pneumoniae in the host is essential for overcoming strain heterogeneity as new therapeutics are developed. Current studies are under way to characterize the KvrA and KvrB regulons in order to gain a fuller understanding of how these proteins influence capsule production and virulence.

## MATERIALS AND METHODS

### Ethical statement.

Mouse experimental procedures were conducted in accordance with the Guide for the Care and Use of Laboratory Animals of the National Institutes of Health. All animal studies were approved by the Institutional Animal Care and Use Committee of the University of North Carolina (UNC) at Chapel Hill. All efforts were made to minimize suffering. Animals were monitored daily following inoculation and were euthanized upon exhibiting signs of morbidity.

### Bacterial strains and culture conditions.

Bacterial strains and plasmids used in this study are described in Table 1. The wild-type K. pneumoniae strains used in this study included KPPR1S (31), a streptomycin-resistant (Strep^r^) derivative of KPPR1 (a rifampin-resistant [Rif^r^] derivative of ATCC 43816 [77]); NTUH-K2044, a K1 isolate from a liver abscess (47); and MKP103, an ST258 carbapenem-sensitive derivative of KPNIH1 (49, 50). All strains were grown aerobically in Luria-Bertani (LB) medium at 37°C. The following antibiotics were added to the media as appropriate at the indicated concentrations: kanamycin (Kan; 50 µg/ml [Kan_50_]), Rif (30 µg/ml [Rif_30_]), and Strep (500 µg/ml).

**TABLE 1:** Bacterial strains and plasmids used in this work

Strain or plasmid	Description^a^	Source or reference
Strains		
* *E. coli DH5α	F^−^ ϕ80d*lacZ*ΔM15 Δ(*lacZYA-argF*)*U169 deoP recA1 endA1 hsdR17* (r_K_^−^ m_K_^−^)	Invitrogen
* *E. coli S17-1λ*pir*	*recA thi pro hsdR hsdM*^*+*^ RP4-2-Tc::Mu::Km Tn*7* λ*pir* (lysogen); Tp^r^ Strep^r^	83
* *K. pneumoniae KPPR1	Rif^r^ derivative of ATCC 43816	77
* *K. pneumoniae KPPR1S	Strep^r^ derivative of KPPR1	31
* *K. pneumoniae VK060	KPPR1 *manC*::*Tn5*:*km*	77
* *K. pneumoniae VK279	KPPR1S *0496*::*pKAS46*	This work
* *K. pneumoniae VK280	KPPR1S *4504*::*pKAS46*	This work
* *K. pneumoniae VK277	KPPR1S Δ*kvrA* (VK055_0496)	This work
* *K. pneumoniae VK410	KPPR1S Δ*kvrB* (VK055_4504)	This work
* *K. pneumoniae VK332	KPPR1S Δ*marR* (VK055_0870)	This work
* *K. pneumoniae VK278	KPPR1S *kvrA* complemented at native site	This work
* *K. pneumoniae VK417	KPPR1S *kvrB* complemented at native site	This work
* *K. pneumoniae VK322	KPPR1S *4417*::*pKAS46*	This work
* *K. pneumoniae VK323	KPPR1S *4238*::*pKAS46*	This work
* *K. pneumoniae VK324	KPPR1S *1202*::*pKAS46*	This work
* *K. pneumoniae VK325	KPPR1S *2679*::*pKAS46*	This work
* *K. pneumoniae VK326	KPPR1S *4504*::*pKAS46*	This work
* *K. pneumoniae VK327	KPPR1S *1021*::*pKAS46*	This work
* *K. pneumoniae VK328	KPPR1S *1682*::*pKAS46*	This work
* *K. pneumoniae NTUH-K2044	Wild-type K. pneumoniae from liver abscess	47
* *K. pneumoniae VK559	NTUH-K2044 p*0496*::*pKAS46*	This work
* *K. pneumoniae K560	NTUH-K2044 p*4504*::*pKAS46*	This work
* *K. pneumoniae MP103	Carbapenem-sensitive derivative of KPNIH1	50
* *K. pneumoniae VK02	MKP103 *kvrA* mutant (KPNIH1_14625)	50
* *K. pneumoniae VK44	MKP103 *kvrB* mutant (KPNIH1_20355)	50
		
Plasmids		
pKAS46	Kan^r^, *ori*R6K cloning vector	78
p*0496*::*pKAS46*	pKAS46 with an internal fragment from VK055_0496	This work
p*4417*::*pKAS46*	pKAS46 with an internal fragment from VK055_4417	This work
p*4238*::*pKAS46*	pKAS46 with an internal fragment from VK055_4238	This work
p*1202*::*pKAS46*	pKAS46 with an internal fragment from VK055_1202	This work
p*2679*::*pKAS46*	pKAS46 with an internal fragment from VK055_2679	This work
p*4504*::*pKAS46*	pKAS46 with an internal fragment from VK055_4504	This work
p*1021*::*pKAS46*	pKAS46 with an internal fragment from VK055_1021	This work
p*1682*::*pKAS46*	pKAS46 with an internal fragment from VK055_1682	This work
pKAS46Δ*kvrA*	*kvrA* flanking regions in pKAS46	This work
pKAS46Δ*kvrB*	*kvrB* flanking regions in pKAS46	This work
pKAS46 *kvrA*comp	*kvrA* flanking regions and gene in pKAS46	This work
pKAS46 *kvrB*comp	*kvrB* flanking regions and gene in pKAS46	This work
pPROBE_tagless	Kan^r^, *gfp* reporter vector	79
pPROBE_*galF*	*galF* promoter of KPPR1S in pPROBE	This work
pPROBE_*wzi*	*wzi* promoter of KPPR1S in pPROBE	This work
pPROBE_*manC*	*cpsB* promoter of KPPR1S in pPROBE	This work

a Cm^r^, chloramphenicol resistance; Tp^r^, trimethoprim resistance.

### Construction of bacterial mutants.

The primers used for the construction of mutant strains are listed in Table 2. In-frame deletion mutants of *kvrA*, *kvrB*, and *marR* (VK277, VK410, and VK332, respectively) were generated by allelic exchange using pKAS46 as described previously (31, 78). Fragments of ~500 bp upstream and downstream of the targeted gene were amplified by PCR, cloned into pKAS46, and verified by sequencing, and the resulting plasmid was introduced into KPPR1S via conjugation. Transconjugants (Rif^r^/Kan^r^) were streaked onto LB-Strep agar plates to select for clones that had lost the vector sequences. The resulting colonies were screened for Kan sensitivity and then for loss of the targeted gene by PCR.

**TABLE 2:** Primers used in this study

Primer	Sequence^a^ (5′ to 3′)	Description^b^
MP159	TGACTA**GATATC**CGACACTCTTAACCAACAGCT	Δ*0496* 5′ flank F
MP160	TGCATA**TCTAGA**CTCCAGGCTAAAGATTAATTC	Δ*0496* 5′ flank R
MP161	TGCATA**TCTAGA**TGGCGATTCCAATTTCATCTC	Δ*0496* 3′ flank F
MP162	TCGATA**GCGGCCGC**CATGCGGCAATCAGGGCGACG	Δ*0496* 3′ flank R
MP228	TGCATA**TCTAGA**GTGCGCACACCTATAAGCGTA	*manC* promoter F
MP229	CAGTAC**GAATTC**GCTCGCGAGACATCGGCCAGA	*manC* promoter R
MP232	TGCATA**TCTAGA**CGGTAATTGATAATTCATATT	*wzi* promoter F
MP233	CAGTAC**GAATTC**TGGGCTCCCAGGGAGGAAAGC	*wzi* promoter R
MP234	TGCATA**TCTAGA**CTGTACGACTGCGGTATGTGT	*galF* promoter F
TM2	CAGTAC**GAATTC**TTTGTGGCCGGCAGCATATGC	*galF* promoter R
MP251	TGACTA**GATATC**CACGACACCATCAGGATGGCG	Δ*0870* 5′ flank F
MP252	TGCATA**TCTAGA**GCTGGTACTTTTCATAATGGT	Δ*0870* 5′ flank R
MP253	TGCATA**TCTAGA**CTCAAGAAGGTCCTGCCGTAA	Δ*0870* 3′ flank F
MP254	TCGATA**GCGGCCGC**CAAGAGTACGATAGCTGCGGC	Δ*0870* 3′ flank R
MP256	TGACTA**GATATC**GGAACGGTTAAGCAACGCCTC	*4417*::*kan* F
MP257	TGCATA**TCTAGA**CTGACTAAACCTCAGTATGCG	*4417*::*kan* R
MP258	TGACTA**GATATC**CTCCAGCGCCTTCAGACGATG	*4238*::*kan* F
MP259	TGCATA**TCTAGA**GCGGCCAGACCTCGACTGCAG	*4238*::*kan* R
MP260	TGACTA**GATATC**GCTGACGGTGGTGTAATCGCG	*1202*::*kan* F
MP261	TGCATA**TCTAGA**TCAGCGTAATGAACCAGCCGC	*1202*::*kan* R
MP262	TGACTA**GATATC**CGGCGGTCAGCGTCAGCAGGC	*2679*::*kan* F
MP263	TGCATA**TCTAGA**GCCAGTGGCGCGCCATTATTG	*2679*::*kan* R
MP274	TGACTA**GATATC**TCAGCGATACGGGTGGCGTTG	*4504*::*kan* F
MP275	TGCATA**TCTAGA**CCGCCATGAAGATTTCCCGTT	*4504*::*kan* R
MP276	TGACTA**GATATC**GGCGGTCAATCGTCGGCTGAA	*1021*::*kan* F
MP277	TGCATA**TCTAGA**GCAGTCGCTTACGTTTGTCGC	*1021*::*kan* R
MP280	TGACTA**GATATC**GGATCTGTTCAATAAGACCGA	*1682*::*kan* F
MP281	TGCATA**TCTAGA**AAGAGCACGTGGAAGGGTTTC	*1682*::*kan* R
MP285	TGACTA**GATATC**CCGATCGAGCGTGACCAGCGG	Δ*4504* 5′ flank F
MP286	TGCATA**TCTAGA**GAAGGCGTGCTAGCGGACTCT	Δ*4504* 5′ flank R
MP287	TGCATA**TCTAGA**AAACGAACTATCCATTTGGGT	Δ*4504* 3′ flank F
MP288	TCGATA**GCGGCCGC**TCTGCGGGTGTCTGACGGCGC	Δ*4504* 3′ flank R
KW372	TAATCGACCATCGCCTGAAAC	*kvrA* 5′ flank F
KW373	GACGGCTGTTCAATACCGATAG	*kvrA* 3′ flank R
KW374	GCCATGAAGATTTCCCGTTTC	*kvrB* 5′ flank F
KW375	CGTTAATTCCCTGCGCTTTG	*kvrB* 3′ flank R

a Restriction sites are in bold.

b F, forward primer; R, reverse primer.

Insertional disruption mutants (VK279, VK322, VK323, VK324, VK325, VK280, VK327, and VK328) were constructed as follows. A DNA fragment containing an internal portion of the gene of interest was generated by PCR, cloned into pKAS46, and verified by sequencing, and the resulting plasmid was introduced in KPPR1S via conjugation. The resulting Rif^r^/Kan^r^ colonies were selected for experimentation. For NTUH-K2044 *0496*::*pKAS46* and NTUH-K2044 *4504*::*pKAS46*, transconjugants were selected on LB plates with chloramphenicol (25 µg/ml) and Kan_50_. Mutants of *kvrA* and *kvrB* were identified in MKP103 from a library of transposon mutants and were kindly provided by C. Manoil (50).

Chromosomal complementation was done by allelic exchange essentially as described above for in-frame deletions. The plasmids for chromosomal complementation (pKAS46 *kvrA*comp and pKAS46 *kvrB*comp) were generated by amplifying a single fragment that spanned the same upstream and downstream regions as the deletion constructs using the outermost primers but that contained the full coding sequence. The insertions were cloned into pKAS46 and verified by sequencing. Following conjugation and resolution of the vector sequences, the resulting complemented strains were designated VK278 (*kvrA*comp) and VK417 (*kvrB*comp).

### Murine model of pneumonia.

Five-to-8-week-old female C57BL/6 mice (Jackson Laboratories) were anesthetized with 200 µl of a 0.8 mg/ml ketamine–1.3 mg/ml xylazine cocktail and inoculated intranasally (i.n.) with ~2 × 10^4^ CFU/mouse (KPPR1S and NTUH-K2044) or ~2 × 10^7^ CFU/mouse (MKP103) as described previously (31, 77). Total CFU counts for each inoculum were confirmed by plating dilutions onto LB agar. After 24, 48, or 72 hpi, mice were euthanized by a lethal intraperitoneal injection of 200 µl sodium pentobarbital (52 mg/ml). Organs were removed, homogenized in phosphate-buffered saline (PBS), serially diluted, and plated to quantify the CFU level per gram of tissue.

### Histopathology.

Groups of two or three mice were inoculated i.n. as described above. The mice were sacrificed at 72 hpi, and lungs were inflated with 1 ml of 10% neutral buffered formalin for at least 24 h. The lungs were transferred to PBS for 2 h and then immersed in 70% ethyl alcohol and embedded in paraffin. Three 5-µm sections (spaced 200 µm apart) per lung were stained with hematoxylin-eosin (H&E) for examination. Histology services were provided by the University of North Carolina (UNC) Center for Gastrointestinal Biology and Disease Histology Core.

### Flow cytometry and antibodies.

The lungs from K. pneumoniae-infected or mock-infected mice were minced into fine pieces with two surgical steel razor blades and then digested in 5.66 mg collagenase II (Gibco, Carlsbad, CA)–4 ml PBS–5% serum for 1 h at 37°C. Following digestion, the lung homogenates were triturated with an 18-gauge needle (three passages) and then passed through a 100-µm-pore-size filter to generate a single-cell suspension.

For flow cytometry staining, approximately 10^7^ cells were counted from red blood cell (RBC)-lysed lung samples. Each sample was incubated with an anti-CD16/32 antibody cocktail (Fc Block; Tonbo Biosciences, San Diego, CA) for 5 min at room temperature. Then, cellular markers were surface labeled by adding a 1:200 dilution of each antibody for 20 min at 4°C. The following antibodies/dyes were used to immunophenotype the cellular infiltrates: Ly-6C-BV786 (clone HK1.4) and anti-CD64-phycoerythrin (PE) (clone X54-5/7.1) from BioLegend (San Diego, CA); anti-CD45-vf450 (clone 30-F11), anti-CD11b-fluorescein isothiocyanate (FITC) (clone M1/70), anti-F4/80-PE/Cy7 (clone BM8.1), and anti-CD19-rf710 (clone 1D3) from Tonbo Biosciences; and anti-Ly-6G-ef660 (clone RB6-8C5) and ef780 fixable viability dye (used at a 1:100 dilution) from Affymetrix EBioscience (San Diego, CA). Flow cytometry data were analyzed using FlowJo version 9.9.4 software (TreeStar, LLC). Cell populations were determined after gating on CD45^+^ CD19^−^ non-B-cell leukocytes, Ly6G^+^/CD11b^+^ neutrophils, and Ly6C^+^ CD11b^+^ CD64^+^ inflammatory monocytes.

### Adherence and internalization assay.

Bone marrow-derived macrophages (BMDM) were prepared as follows. Femurs from C57BL/6 mice were harvested and rinsed thoroughly with Dulbecco’s modified Eagle’s medium (DMEM; Gibco, Carlsbad, CA) to extract immature bone marrow cells. The marrow cells were then strained through a 70-µM-pore-size filter and collected. For each mouse used, the marrow was resuspended to 60 ml of DMEM supplemented with 10% fetal bovine serum (FBS) and 10% L-929 cell supernatant. The cell suspension was then divided into aliquots and placed in 6 untreated 100-mm-diameter petri dishes (10 ml each). The cells were allowed to differentiate for 6 to 7 days, with feeding occurring every 72 h. To verify differentiation, fluorescence-activated cell sorter (FACS) analysis was used to ensure that the levels of F4/80 and CD11b expression were greater than or equal to 90%.

BMDMs were seeded into 24-well plates at a density of 500,000 cells per well and incubated overnight at 37°C and 5% CO_2_. Bacterial cultures were grown in 2 ml LB with Rif_30_ for 16 to 17 h at 37°C. Prior to the assays, the macrophages were rinsed and fresh DMEM–10% FBS was added. For the adherence assay, cells were pretreated for 1 h with 2 µM cytochalasin D (Sigma, St. Louis, MO) to inhibit internalization of bacteria; this step was omitted for the internalization assays. Macrophages were then inoculated with the desired K. pneumoniae strains at a multiplicity of infection (MOI) of 50. After 1 h, the wells were gently rinsed with sterile phosphate-buffered saline (PBS) three times and then lysed with 0.5% saponin–PBS. The lysate was diluted and plated on LB agar to determine the number of CFU. Data are presented as percentages of the inoculum.

For the internalization assay, cells were seeded and treated as described above, omitting the cytochalasin D treatment. Following a 1-h incubation with K. pneumoniae at an MOI of 50, the cells were rinsed with PBS three times, and then DMEM–10% FBS–200 µg/ml gentamycin (Gibco) was added and the reaction mixture was incubated 30 min to kill extracellular bacteria. Following the gentamicin treatment, the cells were lysed with 0.5% saponin–PBS, diluted, and plated on LB agar to determine the number of CFU. Data are presented as percentages of the inoculum.

### Construction of reporter fusions and measurement of promoter activity.

DNA fragments containing the promoter region of *manC*, *galF*, and *wzi* from KPPR1S were amplified by PCR using Pfu Turbo and cloned into the *gfp* reporter plasmid pPROBE-tagless (79). The resulting plasmids, pPROBE-*manC*, pPROBE-*galF*, and pPROBE-*wzi*, were confirmed by restriction digest analysis and sequencing. All primers used for these clones are listed in Table 2.

To examine capsule gene expression *in vitro*, pPROBE-tagless, pPROBE-*manC*, pPROBE-*galF*, or pPROBE-*wzi* was introduced into KPPR1S, VK277, and VK410 by electroporation. These strains were grown overnight at 37°C in LB–Kan_50_, subcultured to an OD_600_ of 0.2, and grown for 6 h. All strains were assayed in triplicate, and each assay was performed multiple times. Relative fluorescent units (RFU) were measured using a Synergy HT plate reader (Biotek Instruments, Winooski, VT), and the measurements were normalized to the culture OD_600_.

### Mucoviscosity assay.

The mucoviscosity of the capsule was determined using the sedimentation assay as previously described (31, 80). Briefly, overnight cultures grown in LB were subcultured to an OD_600_ of 0.2 in fresh media and grown at 37°C. After 6 h, the cultures were normalized to an OD of 1.0/ml and centrifuged for 5 min at 1,000 × *g*. The supernatant was gently removed without disturbing the pellet for OD_600_ measurement.

### Extraction and quantification of capsule.

Uronic acid content was extracted and quantified as previously described (31). Briefly, cultures were grown for 6 h as described above; 500 µl was mixed with 100 µl 1% zwittergent–100 mM citric acid, incubated for 20 min at 50°C, and centrifuged; and 300 µl of supernatant was precipitated with 1.2 ml 100% ethanol. Following centrifugation, the pellet was dried and resuspended in 200 µl distilled water (dH_2_O), and then 1.2 ml sodium tetraborate–concentrated H_2_SO_4_ was added and the reaction mixture was subjected to vortex mixing, boiled for 5 min, and placed on ice for 10 min. Uronic acid was detected by addition of 20 µl of 0.15% 3-phenylphenol (Sigma-Aldrich)–0.5% NaOH. After a 5-min incubation at room temperature, the absorbance at 520 nm was measured. The glucuronic acid content was determined from a standard curve of glucuronate lactone (Sigma-Aldrich, St. Louis, MO) and expressed as micrograms per OD_600_.

### RNA extraction and qRT-PCR analysis.

Overnight cultures of each K. pneumoniae strain were subcultured to an OD_600_ of 0.05 and grown for 2 h in LB (with antibiotics, when necessary). A volume of 3 ml was mixed with 600 µl 1% zwittergent–100 mM citric acid in 2 microcentrifuge tubes and incubated for 10 min at room temperature to facilitate pelleting (81). Total RNA was extracted with Trizol reagent (Invitrogen, Carlsbad, CA) following the manufacturer’s instructions, with the following additional step. A volume of ~100 µl 0.1-mm-diameter silica beads (BioSpec Products, Bartlesville, OK) was added with the Trizol reagent, and the samples were placed in a Precellys 24 homogenator (Bertin Technologies) for 3 min at 5,300 rpm prior to chloroform addition to facilitate lysis. Contaminating DNA was removed with DNA-*free* Turbo using the manufacturer’s rigorous protocol and 1-h incubations. CDNA was synthesized using an iScript cDNA synthesis kit (Bio-Rad, Hercules, CA) with 1 µg RNA as the template in a 20-µl reaction volume, and then the samples were diluted 10-fold with RNase-free dH_2_O. PCR was performed in a 20-µl reaction volume containing 5 µl cDNA, a 500 nM concentration of each primer, and 10 µl SsoAdvance SYBR green Supermix (Bio-Rad) using a CFX96 RealTime system (Bio-Rad). The relative transcript levels for the target genes were normalized to the *gyrB* gene and calculated using the threshold cycle (2^−ΔΔCT^) method (82). The data represent results from an average of 6 biological replicates; 3 of each were grown and processed on separate days.

### Statistical analysis.

Statistical analyses were performed using GraphPad Prism, version 7.0 (San Diego, CA). Mouse infection data were analyzed using the Mann-Whitney test. For all other experiments, ordinary one-way analysis of variance (ANOVA) tests (Dunnett’s multiple-comparison tests) were applied.

## References

[1] Podschun R, Ullmann U 1998 *Klebsiella* spp. as nosocomial pathogens: epidemiology, taxonomy, typing methods, and pathogenicity factors. Clin Microbiol Rev 11:589–603. 9767057 10.1128/cmr.11.4.589 PMC88898

[2] Paczosa MK, Mecsas J 2016 *Klebsiella pneumoniae*: going on the offense with a strong defense. Microbiol Mol Biol Rev 80:629–661. doi:10.1128/MMBR.00078-15. 27307579 PMC4981674

[3] Broberg CA, Palacios M, Miller VL 2014 Klebsiella: a long way to go towards understanding this enigmatic jet-setter. F1000Prime Rep 6:64. doi:10.12703/P6-64. 25165563 PMC4126530

[4] Pope JV, Teich DL, Clardy P, McGillicuddy DC 2011 *Klebsiella pneumoniae* liver abscess: an emerging problem in North America. J Emerg Med 41:e103–e105. doi:10.1016/j.jemermed.2008.04.041. 18993020

[5] Shon AS, Bajwa RPS, Russo TA 2013 Hypervirulent (hypermucoviscous) *Klebsiella pneumoniae*: a new and dangerous breed. Virulence 4:107–118. doi:10.4161/viru.22718. 23302790 PMC3654609

[6] Clegg S, Murphy CN 2016 Epidemiology and virulence of *Klebsiella pneumoniae*. Microbiol Spectr 4:1–17. doi:10.1128/microbiolspec.UTI-0005-2012. 26999397

[7] Follador R, Heinz E, Wyres KL, Ellington MJ, Kowarik M, Holt KE, Thomson NR 2016 The diversity of *Klebsiella pneumoniae* surface polysaccharides. Microb Genomics 2:e000073. doi:10.1099/mgen.0.000073. PMC5320592 28348868

[8] Kawai T 2006 Hypermucoviscosity: an extremely sticky phenotype of *Klebsiella pneumoniae* associated with emerging destructive tissue abscess syndrome. Clin Infect Dis 42:1359–1361. doi:10.1086/503429. 16619145

[9] Yu WL, Ko WC, Cheng KC, Lee HC, Ke DS, Lee CC, Fung CP, Chuang YC 2006 Association between *rmpA* and *magA* genes and clinical syndromes caused by *Klebsiella pneumoniae* in Taiwan. Clin Infect Dis 42:1351–1358. doi:10.1086/503420. 16619144

[10] Ahmad TA, El-Sayed LH, Haroun M, Hussein AA, El Ashry ESH 2012 Development of immunization trials against *Klebsiella pneumoniae*. Vaccine 30:2411–2420. doi:10.1016/j.vaccine.2011.11.027. 22100884

[11] Clements A, Jenney AW, Farn JL, Brown LE, Deliyannis G, Hartland EL, Pearse MJ, Maloney MB, Wesselingh SL, Wijburg OL, Strugnell RA 2008 Targeting subcapsular antigens for prevention of *Klebsiella pneumoniae* infections. Vaccine 26:5649–5653. doi:10.1016/j.vaccine.2008.07.100. 18725260

[12] Munoz-Price LS, Poirel L, Bonomo RA, Schwaber MJ, Daikos GL, Cormican M, Cornaglia G, Garau J, Gniadkowski M, Hayden MK, Kumarasamy K, Livermore DM, Maya JJ, Nordmann P, Patel JB, Paterson DL, Pitout J, Villegas MV, Wang H, Woodford N, Quinn JP 2013 Clinical epidemiology of the global expansion of *Klebsiella pneumoniae* carbapenemases. Lancet Infect Dis 13:785–796. doi:10.1016/S1473-3099(13)70190-7. 23969216 PMC4673667

[13] Cohen SP, Hächler H, Levy SB 1993 Genetic and functional analysis of the multiple antibiotic resistance (*mar*) locus in *Escherichia coli*. J Bacteriol 175:1484–1492. doi:10.1128/jb.175.5.1484-1492.1993. 8383113 PMC193236

[14] Seoane AS, Levy SB 1995 Characterization of MarR, the repressor of the multiple antibiotic resistance (*mar*) operon in *Escherichia coli*. J Bacteriol 177:3414–3419. doi:10.1128/jb.177.12.3414-3419.1995. 7768850 PMC177043

[15] Perera IC, Grove A 2010 Molecular mechanisms of ligand-mediated attenuation of DNA binding by MarR family transcriptional regulators. J Mol Cell Biol 2:243–254. doi:10.1093/jmcb/mjq021. 20716550

[16] Haque MM, Kabir MS, Aini LQ, Hirata H, Tsuyumu S 2009 SlyA, a MarR family transcriptional regulator, is essential for virulence in *Dickeya dadantii* 3937. J Bacteriol 191:5409–5418. doi:10.1128/JB.00240-09. 19542281 PMC2725626

[17] Haque MM, Hirata H, Tsuyumu S 2015 SlyA regulates *motA* and *motB*, virulence and stress-related genes under conditions induced by the PhoP-PhoQ system in *Dickeya dadantii* 3937. Res Microbiol 166:467–475. doi:10.1016/j.resmic.2015.05.004. 26027774

[18] McVicker G, Sun L, Sohanpal BK, Gashi K, Williamson RA, Plumbridge J, Blomfield IC 2011 SlyA protein activates *fimB* gene expression and type 1 fimbriation in *Escherichia coli* K-12. J Biol Chem 286:32026–32035. doi:10.1074/jbc.M111.266619. 21768111 PMC3173223

[19] Wilkinson SP, Grove A 2006 Ligand-responsive transcriptional regulation by members of the MarR family of winged helix proteins. Curr Issues Mol Biol 8:51–62. 16450885

[20] Zhang Y, Luo F, Wu D, Hikichi Y, Kiba A, Igarashi Y, Ding W, Ohnishi K 2015 PrhN, a putative *marR* family transcriptional regulator, is involved in positive regulation of type III secretion system and full virulence of *Ralstonia solanacearum*. Front Microbiol 6:357. doi:10.3389/fmicb.2015.00357. 25972849 PMC4412082

[21] Ellison DW, Lawrenz MB, Miller VL 2004 Invasin and beyond: regulation of *Yersinia* virulence by RovA. Trends Microbiol 12:296–300. doi:10.1016/j.tim.2004.04.006. 15165608

[22] Wang D, Guo C, Gu L, Zhang X 2014 Comparative study of the *marR* genes within the family *Enterobacteriaceae*. J Microbiol 52:452–459. doi:10.1007/s12275-014-3586-2. 24723108

[23] Navarre WW, Halsey TA, Walthers D, Frye J, McClelland M, Potter JL, Kenney LJ, Gunn JS, Fang FC, Libby SJ 2005 Co-regulation of *Salmonella enterica* genes required for virulence and resistance to antimicrobial peptides by SlyA and PhoP/PhoQ. Mol Microbiol 56:492–508. doi:10.1111/j.1365-2958.2005.04553.x. 15813739

[24] Linehan SA, Rytkönen A, Yu XJ, Liu M, Holden DW 2005 SlyA regulates function of *Salmonella* pathogenicity island 2 (SPI-2) and expression of SPI-2-associated genes. Infect Immun 73:4354–4362. doi:10.1128/IAI.73.7.4354-4362.2005. 15972530 PMC1168564

[25] Corbett D, Bennett HJ, Askar H, Green J, Roberts IS 2007 SlyA and H-NS regulate transcription of the *Escherichia coli* K5 capsule gene cluster, and expression of *slyA* in *Escherichia coli* is temperature-dependent, positively autoregulated, and independent of H-NS. J Biol Chem 282:33326–33335. doi:10.1074/jbc.M703465200. 17827501

[26] Lithgow JK, Haider F, Roberts IS, Green J 2007 Alternate SlyA and H-NS nucleoprotein complexes control *hlyE* expression in *Escherichia coli* K-12. Mol Microbiol 66:685–698. doi:10.1111/j.1365-2958.2007.05950.x. 17892462 PMC2156107

[27] Heroven AK, Nagel G, Tran HJ, Parr S, Dersch P 2004 RovA is autoregulated and antagonizes H-NS-mediated silencing of invasin and *rovA* expression in *Yersinia pseudotuberculosis*. Mol Microbiol 53:871–888. doi:10.1111/j.1365-2958.2004.04162.x. 15255899

[28] Cathelyn JS, Ellison DW, Hinchliffe SJ, Wren BW, Miller VL 2007 The RovA regulons of *Yersinia enterocolitica* and *Yersinia pestis* are distinct: evidence that many RovA-regulated genes were acquired more recently than the core genome. Mol Microbiol 66:189–205. doi:10.1111/j.1365-2958.2007.05907.x. 17784909

[29] Revell PA, Miller VL 2000 A chromosomally encoded regulator is required for expression of the *Yersinia enterocolitica inv* gene and for virulence. Mol Microbiol 35:677–685. doi:10.1046/j.1365-2958.2000.01740.x. 10672189

[30] Cathelyn JS, Crosby SD, Lathem WW, Goldman WE, Miller VL 2006 RovA, a global regulator of *Yersinia pestis*, specifically required for bubonic plague. Proc Natl Acad Sci U S A 103:13514–13519. doi:10.1073/pnas.0603456103. 16938880 PMC1569194

[31] Palacios M, Broberg CA, Walker KA, Miller VL 2017 A serendipitous mutation reveals the severe virulence defect of a *Klebsiella pneumoniae fepB* mutant. mSphere 2:e00341-17. doi:10.1128/mSphere.00341-17. 28861522 PMC5566837

[32] Broberg CA, Wu W, Cavalcoli JD, Miller VL, Bachman MA 2014 Complete genome sequence of *Klebsiella pneumoniae* strain ATCC 43816 KPPR1, a rifampin-resistant mutant commonly used in animal, genetic, and molecular biology studies. Genome Announc 2:e00924-14. doi:10.1128/genomeA.00924-14. 25291761 PMC4175196

[33] Weatherspoon-Griffin N, Wing HJ 2016 Characterization of SlyA in *Shigella flexneri* identifies a novel role in virulence. Infect Immun 84:1073–1082. doi:10.1128/IAI.00806-15. 26831468 PMC4807491

[34] Michaux C, Sanguinetti M, Reffuveille F, Auffray Y, Posteraro B, Gilmore MS, Hartke A, Giard JC 2011 SlyA Is a transcriptional regulator involved in the virulence of *Enterococcus faecalis*. Infect Immun 79:2638–2645. doi:10.1128/IAI.01132-10. 21536798 PMC3191995

[35] Lawlor MS, Handley SA, Miller VL 2006 Comparison of the host responses to wild-type and *cpsB* mutant *Klebsiella pneumoniae* infections. Infect Immun 74:5402–5407. doi:10.1128/IAI.00244-06. 16926436 PMC1594822

[36] Cortés G, Borrell N, de Astorza B, Gómez C, Sauleda J, Albertí S 2002 Molecular analysis of the contribution of the capsular polysaccharide and the lipopolysaccharide O side chain to the virulence of *Klebsiella pneumoniae* in a murine model of pneumonia. Infect Immun 70:2583–2590. doi:10.1128/IAI.70.5.2583-2590.2002. 11953399 PMC127904

[37] Cortés G, Alvarez D, Saus C, Albertí S 2002 Role of lung epithelial cells in defense against *Klebsiella pneumoniae* pneumonia. Infect Immun 70:1075–1080. doi:10.1128/IAI.70.3.1075-1080.2002. 11854185 PMC127765

[38] Xiong H, Keith JW, Samilo DW, Carter RA, Leiner IM, Pamer EG 2016 Innate lymphocyte/Ly6C^hi^ monocyte cross talk promotes *Klebsiella pneumoniae* clearance. Cell 165:1–3. doi:10.1016/j.cell.2016.03.013. PMC4842125 27040495

[39] Xiong H, Carter RA, Leiner IM, Tang YW, Chen L, Kreiswirth BN, Pamer EG 2015 Distinct contributions of neutrophils and CCR2 +monocytes to pulmonary clearance of different *Klebsiella pneumoniae* strains. Infect Immun 83:3418–3427. doi:10.1128/IAI.00678-15. 26056382 PMC4534658

[40] Favre-Bonte S, Joly B, Forestier C 1999 Consequences of reduction of *Klebsiella pneumoniae* capsule expression on interactions of this bacterium with epithelial cells. Infect Immun 67:554–561. 9916058 10.1128/iai.67.2.554-561.1999 PMC96354

[41] Fodah RA, Scott JB, Tam H-H, Yan P, Pfeffer TL, Bundschuh R, Warawa JM 2014 Correlation of *Klebsiella pneumoniae* comparative genetic analyses with virulence profiles in a murine respiratory disease model. PLoS One 9:e107394–11. doi:10.1371/journal.pone.0107394. 25203254 PMC4159340

[42] Wu CC, Wang CK, Chen YC, Lin TH, Jinn TR, Lin CT 2014 IscR regulation of capsular polysaccharide biosynthesis and iron-acquisition systems in *Klebsiella pneumoniae* CG43. PLoS One 9:e107812. doi:10.1371/journal.pone.0107812. 25237815 PMC4169559

[43] Lin CT, Chen YC, Jinn TR, Wu CC, Hong YM, Wu WH 2013 Role of the cAMP-dependent carbon catabolite repression in capsular polysaccharide biosynthesis in *Klebsiella pneumoniae*. PLoS One 8:e54430. doi:10.1371/journal.pone.0054430. 23408939 PMC3569464

[44] Hunt JJ, Wang JT, Callegan MC 2011 Contribution of mucoviscosity-associated gene A (*magA*) to virulence in experimental *Klebsiella pneumoniae* endophthalmitis. Invest Ophthalmol Vis Sci 52:6860–6866. doi:10.1167/iovs.11-7798. 21791595 PMC3176006

[45] Lin TL, Yang FL, Yang AS, Peng HP, Li TL, Tsai MD, Wu SH, Wang JT 2012 Amino acid substitutions of MagA in *Klebsiella pneumoniae* affect the biosynthesis of the capsular polysaccharide. PLoS One 7:e46783. doi:10.1371/journal.pone.0046783. 23118860 PMC3485256

[46] Ho JY, Lin TL, Li CY, Lee A, Cheng AN, Chen MC, Wu SH, Wang JT, Li TL, Tsai MD 2011 Functions of some capsular polysaccharide biosynthetic genes in *Klebsiella pneumoniae* NTUH K-2044. PLoS One 6:e21664. doi:10.1371/journal.pone.0021664. 21765903 PMC3134468

[47] Wu KM, Li LH, Yan JJ, Tsao N, Liao TL, Tsai HC, Fung CP, Chen HJ, Liu YM, Wang JT, Fang CT, Chang SC, Shu HY, Liu TT, Chen YT, Shiau YR, Lauderdale TL, Su IJ, Kirby R, Tsai SF 2009 Genome sequencing and comparative analysis of *Klebsiella pneumoniae* NTUH-K2044, a strain causing liver abscess and meningitis. J Bacteriol 191:4492–4501. doi:10.1128/JB.00315-09. 19447910 PMC2704730

[48] Wyres KL, Wick RR, Gorrie C, Jenney A, Follador R, Thomson NR, Holt KE 2016 Identification of *Klebsiella* capsule synthesis loci from whole genome data. Microb Genomics 2:e000102. doi:10.1099/mgen.0.000102. PMC5359410 28348840

[49] Snitkin ES, Zelazny AM, Thomas PJ, Stock F, Program NCS, Henderson DK, Palmore TN, Segre JA 2012 Tracking a hospital outbreak of carbapenem-resistant *Klebsiella pneumoniae* with whole-genome sequencing. Sci Transl Med 4:148ra116. doi:10.1126/scitranslmed.3004129. PMC3521604 22914622

[50] Ramage B, Erolin R, Held K, Gasper J, Weiss E, Brittnacher M, Gallagher L, Manoil C 2017 Comprehensive arrayed transposon mutant library of *Klebsiella pneumoniae* outbreak strain KPNIH1. J Bacteriol 199:e00352-17. doi:10.1128/JB.00352-17. PMC5637181 28760848

[51] Brisse S, Fevre C, Passet V, Issenhuth-Jeanjean S, Tournebize R, Diancourt L, Grimont P 2009 Virulent clones of *Klebsiella pneumoniae*: identification and evolutionary scenario based on genomic and phenotypic characterization. PLoS One 4:e4982. doi:10.1371/journal.pone.0004982. 19319196 PMC2656620

[52] Holt KE, Wertheim H, Zadoks RN, Baker S, Whitehouse CA, Dance D, Jenney A, Connor TR, Hsu LY, Severin J, Brisse S, Cao H, Wilksch J, Gorrie C, Schultz MB, Edwards DJ, Nguyen KV, Nguyen TV, Dao TT, Mensink M, Minh VL, Nhu NTK, Schultsz C, Kuntaman K, Newton PN, Moore CE, Strugnell RA, Thomson NR 2015 Genomic analysis of diversity, population structure, virulence, and antimicrobial resistance in *Klebsiella pneumoniae*, an urgent threat to public health. Proc Natl Acad Sci U S A 112:E3574–E3581. doi:10.1073/pnas.1501049112. PMC4500264 26100894

[53] Rosen DA, Hilliard JK, Tiemann KM, Todd EM, Morley SC, Hunstad DA 2016 *Klebsiella pneumoniae* FimK promotes virulence in murine pneumonia. J Infect Dis 213:649–658. doi:10.1093/infdis/jiv440. 26347570 PMC4721909

[54] Wagner NJ, Lin CP, Borst LB, Miller VL 2013 YaxAB, a *Yersinia enterocolitica* pore-forming toxin regulated by RovA. Infect Immun 81:4208–4219. doi:10.1128/IAI.00781-13. 24002058 PMC3811828

[55] Ye P, Rodriguez FH, Kanaly S, Stocking KL, Schurr J, Schwarzenberger P, Oliver P, Huang W, Zhang P, Zhang J, Shellito JE, Bagby GJ, Nelson S, Charrier K, Peschon JJ, Kolls JK 2001 Requirement of interleukin 17 receptor signaling for lung CXC chemokine and granulocyte colony-stimulating factor expression, neutrophil recruitment, and host defense. J Exp Med 194:519–527. doi:10.1084/jem.194.4.519. 11514607 PMC2193502

[56] Domenico P, Salo RJ, Cross AS, Cunha BA 1994 Polysaccharide capsule-mediated resistance to opsonophagocytosis in *Klebsiella pneumoniae*. Infect Immun 62:4495–4499. 7927714 10.1128/iai.62.10.4495-4499.1994 PMC303135

[57] Wang ZC, Liu CJ, Huang YJ, Wang YS, Peng HL 2015 PecS regulates the urate-responsive expression of type 1 fimbriae in *Klebsiella pneumoniae* CG43. Microbiology 161:2395–2409. doi:10.1099/mic.0.000185. 26385366

[58] Stahlhut SG, Struve C, Krogfelt KA, Reisner A 2012 Biofilm formation of *Klebsiella pneumoniae* on urethral catheters requires either type 1 or type 3 fimbriae. FEMS Immunol Med Microbiol 65:350–359. doi:10.1111/j.1574-695X.2012.00965.x. 22448614 PMC3410544

[59] Bachman MA, Breen P, Deornellas V, Mu Q, Zhao L, Wu W, Cavalcoli JD, Mobley HLT 2015 Genome-wide identification of *Klebsiella pneumoniae* fitness genes during lung infection. MBio 6:e00775-15. doi:10.1128/mBio.00775-15. 26060277 PMC4462621

[60] Struve C, Bojer M, Krogfelt KA 2009 Identification of a conserved chromosomal region encoding *Klebsiella pneumoniae* type 1 and type 3 fimbriae and assessment of the role of fimbriae in pathogenicity. Infect Immun 77:5016–5024. doi:10.1128/IAI.00585-09. 19703972 PMC2772557

[61] Murphy CN, Mortensen MS, Krogfelt KA, Clegg S 2013 Role of *Klebsiella pneumoniae* type 1 and type 3 fimbriae in colonizing silicone tubes implanted into the bladders of mice as a model of catheter-associated urinary tract infections. Infect Immun 81:3009–3017. doi:10.1128/IAI.00348-13. 23753626 PMC3719564

[62] Yeh KM, Chiu SK, Lin CL, Huang LY, Tsai YK, Chang JC, Lin JC, Chang FY, Siu LK 2016 Surface antigens contribute differently to the pathophysiological features in serotype K1 and K2 *Klebsiella pneumoniae* strains isolated from liver abscesses. Gut Pathog 8:4. doi:10.1186/s13099-016-0085-5. 26893615 PMC4758166

[63] Lee CH, Chang CC, Liu JW, Chen RF, Yang KD 2014 Sialic acid involved in hypermucoviscosity phenotype of *Klebsiella pneumoniae* and associated with resistance to neutrophil phagocytosis. Virulence 5:673–679. doi:10.4161/viru.32076. 25098744 PMC4139408

[64] Moranta D, Regueiro V, March C, Llobet E, Margareto J, Larrarte E, Garmendia J, Bengoechea JA 2010 *Klebsiella pneumoniae* capsule polysaccharide impedes the expression of beta-defensins by airway epithelial cells. Infect Immun 78:1135–1146. doi:10.1128/IAI.00940-09. 20008534 PMC2825953

[65] Woodward L, Naismith JH 2016 Bacterial polysaccharide synthesis and export. Curr Opin Struct Biol 40:81–88. doi:10.1016/j.sbi.2016.07.016. 27544430

[66] Rahn A, Beis K, Naismith JH, Whitfield C 2003 A novel outer membrane protein, Wzi, is involved in surface assembly of the *Escherichia coli* K30 group 1 capsule. J Bacteriol 185:5882–5890. doi:10.1128/JB.185.19.5882-5890.2003. 13129961 PMC193962

[67] Bushell SR, Mainprize IL, Wear MA, Lou H, Whitfield C, Naismith JH 2013 Wzi Is an outer membrane lectin that underpins group 1 capsule assembly in *Escherichia coli*. Structure 21:844–853. doi:10.1016/j.str.2013.03.010. 23623732 PMC3791409

[68] Fang CT, Chuang YP, Shun CT, Chang SC, Wang JT 2004 A novel virulence gene in *Klebsiella pneumoniae* strains causing primary liver abscess and septic metastatic complications. J Exp Med 199:697–705. doi:10.1084/jem.20030857. 14993253 PMC2213305

[69] Allen PM, Fisher D, Saunders JR, Hart CA 1987 The role of capsular polysaccharide K21b of *Klebsiella* and of the structurally related colanic-acid polysaccharide of *Escherichia coli* in resistance to phagocytosis and serum killing. J Med Microbiol 24:363–370. doi:10.1099/00222615-24-4-363. 3320374

[70] Llobet E, Tomás JM, Bengoechea JA 2008 Capsule polysaccharide is a bacterial decoy for antimicrobial peptides. Microbiology 154:3877–3886. doi:10.1099/mic.0.2008/022301-0. 19047754

[71] Libby SJ, Goebel W, Ludwig A, Buchmeier N, Bowe F, Fang FC, Guiney DG, Songer JG, Heffron F 1994 A cytolysin encoded by *Salmonella* is required for survival within macrophages. Proc Natl Acad Sci U S A 91:489–493. doi:10.1073/pnas.91.2.489. 8290552 PMC42974

[72] Del Castillo I, González-Pastor JE, San Millán JL, Moreno F 1991 Nucleotide sequence of the *Escherichia coli* regulatory gene *mprA* and construction and characterization of *mprA*-deficient mutants. J Bacteriol 173:3924–3929. doi:10.1128/jb.173.12.3924-3929.1991. 1840583 PMC208030

[73] Stoebel DM, Free A, Dorman CJ 2008 Anti-silencing: overcoming H-NS-mediated repression of transcription in Gram-negative enteric bacteria. Microbiology 154:2533–2545. doi:10.1099/mic.0.2008/020693-0. 18757787

[74] Ellison DW, Miller VL 2006 H-NS represses *inv* transcription in *Yersinia enterocolitica* through competition with RovA and interaction with YmoA. J Bacteriol 188:5101–5112. doi:10.1128/JB.00862-05. 16816182 PMC1539963

[75] Navarre WW, McClelland M, Libby SJ, Fang FC 2007 Silencing of xenogeneic DNA by H-NS-facilitation of lateral gene transfer in bacteria by a defense system that recognizes foreign DNA. Genes Dev 21:1456–1471. doi:10.1101/gad.1543107. 17575047

[76] Hommais F, Krin E, Laurent-Winter C, Soutourina O, Malpertuy A, Le Caer JP, Danchin A, Bertin P 2001 Large-scale monitoring of pleiotropic regulation of gene expression by the prokaryotic nucleoid-associated protein, H-NS. Mol Microbiol 40:20–36. 11298273 10.1046/j.1365-2958.2001.02358.x

[77] Lawlor MS, Hsu J, Rick PD, Miller VL 2005 Identification of *Klebsiella pneumoniae* virulence determinants using an intranasal infection model. Mol Microbiol 58:1054–1073. doi:10.1111/j.1365-2958.2005.04918.x. 16262790

[78] Skorupski K, Taylor RK 1996 Positive selection vectors for allelic exchange. Gene 169:47–52. doi:10.1016/0378-1119(95)00793-8. 8635748

[79] Miller WG, Leveau JH, Lindow SE 2000 Improved *gfp* and *inaZ* broad-host-range promoter-probe vectors. Mol Plant Microbe Interact 13:1243–1250. doi:10.1094/MPMI.2000.13.11.1243. 11059491

[80] Ares MA, Fernández-Vázquez JL, Rosales-Reyes R, Jarillo-Quijada MD, Bargen von K, Torres J, González-y-Merchand JA, Alcántar-Curiel MD, De la Cruz MA 2016 H-NS nucleoid protein controls virulence features of Klebsiella pneumoniae by regulating the expression of type 3 pili and the capsule polysaccharide. Front Cell Infect Microbiol 6:13. doi:10.3389/fcimb.2016.00013. 26904512 PMC4746245

[81] Domenico P, Marx JL, Schoch PE, Cunha BA 1992 Rapid plasmid DNA isolation from mucoid Gram-negative bacteria. J Clin Microbiol 30:2859–2863. 1333482 10.1128/jcm.30.11.2859-2863.1992 PMC270542

[82] Livak KJ, Schmittgen TD 2001 Analysis of relative gene expression data using real-time quantitative PCR and the 2-ΔΔCT method. Methods 25:402–408. doi:10.1006/meth.2001.1262. 11846609

[83] Miller VL, Mekalanos JJ 1988 A novel suicide vector and its use in construction of insertion mutations: osmoregulation of outer membrane proteins and virulence determinants in *Vibrio cholerae* requires *toxR*. J Bacteriol 170:2575–2583. doi:10.1128/jb.170.6.2575-2583.1988. 2836362 PMC211174

